# Can handgrip strength alone detect individuals living with frailty according to the Clinical Frailty Scale?

**DOI:** 10.1590/1806-9282.20240237

**Published:** 2025-05-02

**Authors:** Serdar Ceylan, Yelda Ozturk, Merve Guner, Arzu Okyar Bas, Meltem Koca, Cafer Balci, Burcu Balam Dogu, Mustafa Cankurtaran, Meltem Gulhan Halil

**Affiliations:** 1 Hacettepe University, Faculty of Medicine, Department of Internal Medicine, Division of Geriatrics – Ankara, Turkey.

**Keywords:** Hand strength, Frailty, Sarcopenia, Older adults

## Abstract

**OBJECTIVE:**

Several studies have been conducted to determine handgrip strength cutoffs that can identify people living with frailty. However, the handgrip strength cutoff value, which can detect individuals living with frailty based on the Clinical Frailty Scale, has not been determined before. The aim of this study was to investigate the capacity of handgrip strength to detect individuals living with frailty by using Clinical Frailty Scale as a reference scale.

**METHODS:**

This retrospective study was carried out by including patients who applied to the geriatric outpatient clinic of a university hospital. A comprehensive geriatric assessment was performed on all patients. Level 4 and above were considered as living with frailty according to Clinical Frailty Scale. Receiver operating characteristic curve analysis was performed to determine the handgrip strength cutoff values for predicting individuals living with frailty.

**RESULTS:**

The median age of 742 patients included in this study was 72.0 years (25p-75p: 68.0-77.0), of which 59.3% (n=440) were female and 49.3% (n=366) were living with frailty. The median Clinical Frailty Scale level was 3.0 (25p-75p: 3.0-4.0). According to the results of binary logistic regression analysis, age, sex, and handgrip strength displayed a statistically significant relationship with frailty (p<0001, p=0.001, and p<0.001, respectively). As a result of the receiver operating characteristic analysis performed to determine the handgrip strength cutoff values that predict frailty, cutoff values of 16 kg for females and 26.7 kg for males were identified. The area under the curve values for females and males were 0.679 (p<0.001) and 0.790 (p<0.001), respectively.

**CONCLUSION:**

Handgrip strength can be used alone as a predictor to identify individuals living with frailty.

## INTRODUCTION

Life expectancy has nearly doubled as a result of advances in modern medicine and public health. The increase in life expectancy has generated several issues. Chronic diseases, cognitive impairments, mental disorders, falls, and other issues have appeared more frequently^1^. These issues have paved the way for the emergence of many innovative markers. One of them is the concept of frailty, which is used to evaluate and manage older adult patients more accurately. Frailty arises from an interruption in the harmonious interaction of biological, genetic, functional, cognitive, psychological, and socioeconomic dimensions. Although there is no formal definition, the general consensus is that it is the accumulation of deficits or losses in physical functions^2^. Frailty is associated with many adverse health outcomes, including the need for care, falls, hospitalization, and mortality. It is consequently critical to detect the frailty status^3^.

Many scales have been designed to assess frailty. There is, however, no gold standard scale^4^. The Clinical Frailty Scale (CFS) is one of the most frequently used scales in frailty screening. It was developed in the second phase of the Canadian Study of Health and Aging^5^. Grading is made from 1 to 9. Its usability in the aged population in Türkiye was examined in a study by Aşık et al., and it was identified as a fast, reliable, and valid frailty screening tool for older adults in the Turkish population^6^. The CFS is widely used by non-geriatrician health personnel due to its advantages of easy application, short-time performance, no need for devices, and success in predicting health outcomes in different patient groups^7^.

Handgrip strength (HGS) describes a physical function used in the screening of sarcopenia, with different cutoff points for females and males and differences in the cutoff points according to the genetic characteristics of the populations, and it is measured with the help of an auxiliary device^8^. Although lower HGS values are associated with a risk of living with frailty (LWF)^9^, it has been questioned whether HGS alone can be used to identify patients LWF^10^. Several researches have been conducted to determine the HGS cutoffs that can identify individuals LWF^11,12^. However, the HGS cutoff value, which can detect individuals LWF based on CFS, has not been determined before. Hence, the present study aims to investigate the capacity of HGS to detect individuals LWF by using CFS as a reference scale.

## METHODS

### Participants

This retrospective study was carried out by including patients who applied to the geriatric outpatient clinic of a university hospital. The inclusion criteria were defined as being 65 years of age or older and having CFS and HGS values recorded in the hospital automation system. Patient demographic information (age, sex, educational level, marital status, and with whom they lived) and other details including presence of chronic diseases, dosage of medications taken, and comprehensive geriatric assessment results were recorded. The usage of five or more medicines per day was considered as polypharmacy, and the presence of two or more chronic diseases together was considered as multimorbidity.

### Clinical Frailty Scale

The CFS was developed in the second phase of the Canadian Study of Health and Aging. Individuals are evaluated by taking into account their activities of daily living (ADL), mobility, disease symptoms, cognitive status, and life expectancy. It is a 9-point scale. Level 4 and above are considered as LWF. Levels 1-9 are graded as very fit, fit, managing well, living with very mild frailty, living with mild frailty, living with moderate frailty, living with severe frailty, living with very severe frailty, and terminally ill. The scale's validity and reliability in the Turkish geriatric population have been proven by Aşık et al.^6^.

### Handgrip strength

The HGS was measured using a hand dynamometer (Grip Strength Takei dynamometer, Niigata City, Japan). The measurement was performed three times with the dominant hand, the elbow flexion at 90°, and a neutrally rotated forearm when the patient was in the sitting position. The highest value was recorded.

### Ethical approval

The study was approved by the Non-interventional Research Ethics Board of the Faculty of Medicine, University (blinded for review) (decision number: [blinded for review]). The authors declared that the study was conducted in accordance with the Declaration of Helsinki and the ethical standards of Türkiye were followed.

### Statistical analysis

Data were analyzed using the Statistical Package for the Social Sciences (SPSS) software version 24. Categorical variables were expressed as numbers and percentages, and numerical variables were expressed as mean and standard deviation or median and percentiles (p) according to the normal distribution condition. According to the normal distribution, comparisons were made with Student's t-test or Mann-Whitney U test for numerical variables and chi-square test for categorical variables. Binary logistic regression analysis was performed to determine the variables that affect frailty independently. Receiver operating characteristic (ROC) curve analysis was carried out to determine the HGS cutoff values for predicting individuals LWF. Sensitivity, specificity, positive predictive value (PPV), and negative predictive value (NPV) were calculated. A p-value of 0.05 was deemed statistically significant.

## RESULTS

The median age of 742 patients included in the study was 72.0 years (25p–75p: 68.0–77.0), of which 59.3% (n=440) were female, 78.4% (n=582) were living with multimorbidity, 63.2% (n=469) had polypharmacy, and 49.3% (n=366) were LWF. The median CFS level was 3.0 (25p–75p: 3.0–4.0). There was a statistically significant difference among demographics, clinical characteristics, and geriatric assessment results such as age, educational level, multimorbidity, polypharmacy, Katz ADL, Lawton-Brody Instrumental ADL, Mini Nutritional Assessment-Short Form, Mini-Mental State Examination, Strength, Assistance with Walking, Rising from a Chair, Climbing Stairs, and Falls, HGS, and falls between patients LWF and those not LWF (p=0.004 for multimorbidity and p<0.001 for other variables) (Table 1). According to the results of the binary logistic regression analysis, age, sex, and HGS displayed a statistically significant relationship with frailty (p<0001, p=0.001, and p<0.001, respectively) (Table 2).

**Table 1:** Patient demographics, clinical characteristics, and comprehensive geriatric assessment results.

		Total (n=742)	LWF (n=366, 49.3%)	Robust (n=376, 50.7%)	p
Age* (years)		72.0 (68.0-77.0)	74.5 (70.0-80.0)	70.0 (67.0-74.0)	<0.001
Sex (female)		440 (59.3)	224 (61.2)	216 (57.4)	0.30
Education (<8 years)		367 (49.5)	217 (59.3)	150 (39.9)	<0.001
Married		392 (52.8)	196 (53.6)	196 (52.1)	0.70
Living alone		109 (14.7)	55 (15.0)	54 (14.4)	0.80
Smoking		173 (23.3)	85 (23.2)	88 (23.4)	0.95
Multimorbidity		582 (78.4)	303 (82.8)	279 (74.2)	0.004
Polypharmacy		469 (63.2)	261 (71.3)	208 (55.3)	<0.001
Comprehensive geriatric assessment					
	Katz ADL*	6.0 (5.0-6.0)	6.0 (5.0-6.0)	6.0 (6.0-6.0)	<0.001
	Lawton-Brody Instrumental ADL*	8.0 (7.0-8.0)	8.0 (5.0-8.0)	8.0 (8.0-8.0)	<0.001
	Mini Nutritional Assessment-Short Form*	14.0 (12.0-14.0)	13.0 (10.0-14.0)	14.0 (13.0-14.0)	<0.001
	Mini-Mental State Examination*	28.0 (25.0-29.0)	26.0 (23.0-28.0)	29.0 (27.0-30.0)	<0.001
	SARC-F*	1.0 (0.0-3.0)	3.0 (1.0-5.0)	0.0 (0.0-1.0)	<0.001
HGS# (kg)					
	Female	17.6±5.1	16.1±4.7	19.2±5.0	<0.001
	Male	26.2±7.4	21.4±6.9	29.5±6.2	<0.001
	Falls	182 (24.5)	113 (30.9)	69 (18.4)	<0.001

* Median, 25-75 percentiles.

# Mean±standard deviation.

A CFS score ≥4 is considered LWF. LWF: living with frailty; ADL: activities of daily living; HGS: handgrip strength; CFS: Clinical Frailty Scale; SARC-F: Strength, Assistance with Walking, Rising from a Chair, Climbing Stairs, and Falls.

**Table 2:** Binary logistic regression analysis of variables affecting frailty.

	Total			Female			Male		
	OR	95%CI	p	OR	95%CI	p	OR	95%CI	P
Age (per year)	1.09	1.06-1.12	<0.001	1.11	1.07-1.16	<0.001	1.06	1.01-1.10	0.02
Sex (female)	2.11	1.38-3.23	0.001	–	–	–	–	–	–
Multimorbidity	1.30	0.88-1.93	0.19	0.96	0.57-1.60	0.86	2.01	1.06-3.78	0.03
HGS (per kg)	0.89	0.86-0.92	<0.001	0.91	0.87-0.95	<0.001	0.88	0.84-0.91	<0.001

CFS score ≥4 is accepted as LWF. LWF: living with frailty; OR: odds ratio; CI: confidence interval; CFS: Clinical Frailty Scale; HGS: handgrip strength.

**Figure 1:**
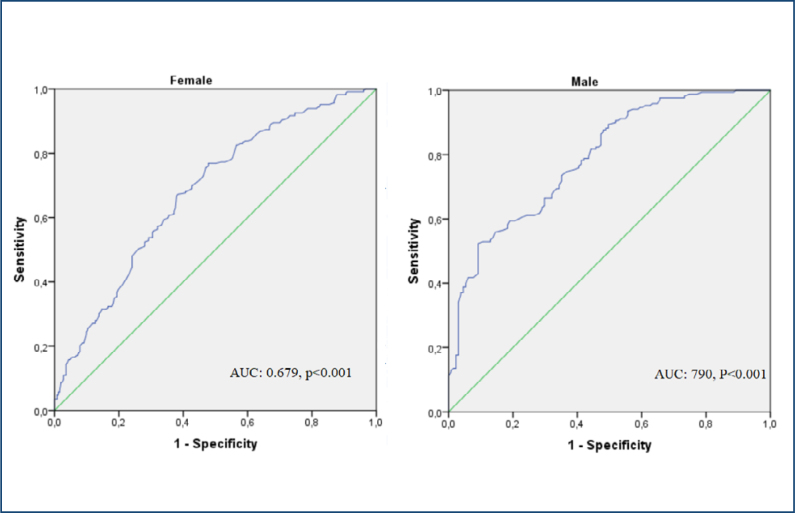
Receiver operating characteristic curve analysis according to sex. ROC: receiver operating characteristic, AUC: area under the curve.

The ROC curve analysis performed to determine the HGS cutoff values that predict frailty revealed cutoff values of 16 kg for females and 26.7 kg for males. The area under the curve (AUC) values for females and males were 0.679 (p<0.001) and 0.790 (p<0.001), respectively (Figure 1). The sensitivity, specificity, PPV, and NPV values are summarized in Table 3.

**Table 3:** Receiver operating characteristic curve analysis results.

	AUC	HGS cutoff (kg)	Sensitivity (%)	Specificity (%)	PPV (%)	NPV (%)
Total (95%CI)	–	–	57.1 (51.9-62.2)	73.9 (69.2-78.3)	68.1 (62.5-73.3)	63.9 (59.2-68.4)
Female (95%CI)	0.679	16.0	50.9 (44.2-57.6)	76.9 (70.7-82.3)	69.5 (61.9-76.5)	60.1 (54.1-66.0)
Male (95%CI)	0.790	26.7	66.9 (58.5-74.6)	70.0 (62.3-77.0)	66.4 (58.1-74.1)	70.4 (62.7-77.4)

AUC: area under the curve; HGS: handgrip strength; PPV: positive predictive value; NPV: negative predictive value; CI: confidence interval; ROC: receiver operating characteristic.

## DISCUSSION

The capacity of HGS, one of the indicators of physical performance in older adults, in predicting individuals LWF was evaluated using CFS as a reference scale. The HGS cutoff points for predicting frailty have been identified to be 16 kg for females and 26.7 kg for males. The HGS is capable of detecting individuals LWF according to CFS, which not only provides a physical assessment but also allows the patient to be evaluated from several aspects.

HGS is one of the measures used to assess an individual's physical performance. It is used in the screening of sarcopenia according to the European Working Group on Sarcopenia in Older People criteria and is defined as probable sarcopenia if HGS cutoff value is low^8^. While its main role is in sarcopenia, the association between a decrease in HGS and other adverse health outcomes has piqued researchers’ interest. Its relationship with a variety of outcomes has been studied across a wide range of patient categories. The risks of cardiometabolic diseases, cognitive impairments, disabilities, hospitalizations, prolonged length of hospital stays, LWF, and mortalities increase with a decrease in HGS^13–15^. It is also part of the locomotory and vitality components of intrinsic capacity (IC)^16^. In addition, considering its relationship with cognition and mood, HGS is an indicator that can be used to monitor overall IC^17^. Therefore, HGS serves as a crucial guide for clinicians in patient follow-up.

Frailty is typically viewed as a complex entity comprising physical, cognitive, psychological, and social components. There is no fully agreed-upon definition^2^. Essentially, three models are used to define frailty: rule-based^18^, summing the number of deficits (deficit accumulation)^19^, and clinical judgment^5^. A typical example of the rule-based frailty definition is the Fried frailty phenotype (FFP). One of the characteristics of individuals LWF in FFP is low HGS^18^. One of the shortfalls in the deficit accumulation model could be a decrease in HGS^20^. In the clinical judgment model, frailty is specified according to the clinician's decision as a result of the medical history and clinical examination. The CFS is one of the most popular examples of clinical judgment models. The final decision is made by considering conditions, such as mobility, disease symptoms, ADL, cognition, and life expectancy^5^. Although HGS is not one of the elements covered in CFS, there is a significant association between CFS and HGS, particularly in females. The lower the HGS, the higher the CFS level and the risk of LWF^21^. In the present study, CFS and HGS were associated in both sexes independently of other variables.

Although HGS and CFS scores are closely related, HGS cutoff points that can predict LWF have not been determined according to CFS. However, some cutoff points are specified according to other frailty scales. For instance, the HGS cutoff point in the FFP is 20% lower than the values assigned by age and body mass index^18^. It has been stated that different cutoff values can be used in different races due to the difference in the muscle structure between races^22^. In addition, the capacity of HGS alone to detect individuals LWF has been tested in various studies. When FFP is used as a reference scale in older Chinese adults, the HGS cutoff values as a single predictor, which can detect individuals LWF, were 18 kg for females and 28 kg for males^11^. In a study conducted on female rheumatoid arthritis patients, the HGS cutoff value for predicting individuals LWF using the Kihon Checklist as a reference scale was 17 kg^23^. In a Turkish validation study of FFP, the HGS cutoff values were 13.6 kg for females and 27.7 kg for males^24^. In the present study, similar to previous studies, the HGS cutoff values were defined as 16 kg for females and 26.7 kg for males.

### Limitations and strengths

This study has some limitations. The association of HGS with adverse health outcomes could not be assessed since the study was cross-sectional in nature. Different clinicians have applied CFS, and this may have caused an operator bias. The study's main strength is that the HGS, which is a physical measurement, has the capacity of detecting individuals LWF in a large sample size and it uses a reference scale that provides a multi-dimensional evaluation of individuals and also includes the clinician's opinion. Another strength is that all patients in the present study underwent a comprehensive geriatric assessment.

## CONCLUSION

HGS can be used alone to identify individuals LWF. Prospective studies are needed to ascertain the capacity of determined HGS cutoff values in predicting adverse health outcomes.

## References

[1] Beard JR, Officer A, Carvalho IA, Sadana R, Pot AM, Michel JP (2016). The world report on ageing and health: a policy framework for healthy ageing. Lancet.

[2] Pilotto A, Custodero C, Maggi S, Polidori MC, Veronese N, Ferrucci L (2020). A multidimensional approach to frailty in older people. Ageing Res Rev.

[3] Walston J, Hadley EC, Ferrucci L, Guralnik JM, Newman AB, Studenski SA (2006). Research agenda for frailty in older adults: toward a better understanding of physiology and etiology: summary from the American Geriatrics Society/National Institute on Aging Research Conference on Frailty in Older Adults. J Am Geriatr Soc.

[4] Buta BJ, Walston JD, Godino JG, Park M, Kalyani RR, Xue QL (2016). Frailty assessment instruments: systematic characterization of the uses and contexts of highly-cited instruments. Ageing Res Rev.

[5] Rockwood K, Song X, MacKnight C, Bergman H, Hogan DB, McDowell I (2005). A global clinical measure of fitness and frailty in elderly people. CMAJ.

[6] Aşık Z, Kılınç Ş, Kurşun Ö, Özen M (2022). Validation of the Clinical Frailty Scale version 2.0 in Turkish older patients. Geriatr Gerontol Int.

[7] Moreno-Ariño M, Torrente Jiménez I, Cartanyà Gutiérrez A, Oliva Morera JC, Comet R (2020). Assessing the strengths and weaknesses of the Clinical Frailty Scale through correlation with a frailty index. Aging Clin Exp Res.

[8] Cruz-Jentoft AJ, Bahat G, Bauer J, Boirie Y, Bruyère O, Cederholm T (2019). Sarcopenia: revised European consensus on definition and diagnosis. Age Ageing.

[9] Marano L, Carbone L, Poto GE, Gambelli M, Nguefack Noudem LL, Grassi G (2022). Handgrip strength predicts length of hospital stay in an abdominal surgical setting: the role of frailty beyond age. Aging Clin Exp Res.

[10] Syddall H, Cooper C, Martin F, Briggs R, Aihie Sayer A (2003). Is grip strength a useful single marker of frailty?. Age Ageing.

[11] Auyeung TW, Lee JS, Leung J, Kwok T, Woo J (2014). The selection of a screening test for frailty identification in community-dwelling older adults. J Nutr Health Aging.

[12] Velghe A, Buyser S, Noens L, Demuynck R, Petrovic M (2016). Hand grip strength as a screening tool for frailty in older patients with haematological malignancies. Acta Clin Belg.

[13] McGrath R, Johnson N, Klawitter L, Mahoney S, Trautman K, Carlson C (2020). What are the association patterns between handgrip strength and adverse health conditions? A topical review. SAGE Open Med.

[14] Kim J (2021). Handgrip strength to predict the risk of all-cause and premature mortality in Korean adults: a 10-year cohort study. Int J Environ Res Public Health.

[15] Soysal P, Hurst C, Demurtas J, Firth J, Howden R, Yang L (2021). Handgrip strength and health outcomes: umbrella review of systematic reviews with meta-analyses of observational studies. J Sport Health Sci.

[16] George PP, Lun P, Ong SP, Lim WS (2021). A rapid review of the measurement of intrinsic capacity in older adults. J Nutr Health Aging.

[17] Arokiasamy P, Selvamani Y, Jotheeswaran AT, Sadana R (2021). Socioeconomic differences in handgrip strength and its association with measures of intrinsic capacity among older adults in six middle-income countries. Sci Rep.

[18] Fried LP, Tangen CM, Walston J, Newman AB, Hirsch C, Gottdiener J (2001). Frailty in older adults: evidence for a phenotype. J Gerontol A Biol Sci Med Sci.

[19] Rockwood K, Mitnitski AB, MacKnight C (2002). Some mathematical models of frailty and their clinical implications. Rev Clin Gerontol.

[20] Brown JD, Alipour-Haris G, Pahor M, Manini TM (2020). Association between a deficit accumulation frailty index and mobility outcomes in older adults: secondary analysis of the lifestyle interventions and independence for elders (LIFE) study. J Clin Med.

[21] Spiegowski D, Metzger L, Jain A, Inchiosa MA, Weber G, Abramowicz AE (2022). The utility of grip strength as a simplified measure of frailty in the older adult in the preoperative clinic. Cureus.

[22] Bahat G, Kilic C, Altinkaynak M, Akif Karan M (2020). Comparison of standard versus population-specific handgrip strength cut-off points in the detection of probable sarcopenia after launch of EWGSOP2. Aging Male.

[23] Sobue Y, Suzuki M, Ohashi Y, Koshima H, Okui N, Funahashi K (2022). Validation of grip strength as a measure of frailty in rheumatoid arthritis. Sci Rep.

[24] Varan HD, Deniz O, Çöteli S, Doğrul RT, Kızılarslanoğlu MC, Göker B (2022). Validity and reliability of Fried frailty phenotype in Turkish population. Turk J Med Sci.

